# 

**Published:** 1861-12

**Authors:** 


☞ THE FOURTH ANNUAL SESSION OF THE INDIANA STATE DENTAL ASSOCIATION is hereby announced to be held in the City of Lafayette, commencing at 2 o'clock, P. M., on the *second Tuesday in January*, 1862.

In making the above announcement, the Secretary begs leave, most respectfully, to urge upon each and every member the importance of a full and prompt attendance at our coming meeting.

Causes not necessary to refer to rendered it advisable to postpone the convocation of the semi-annual meeting ordered in July last, of which due notice was given by Circular. Much business relating to the organization and the interests of the profession in the State, has thus accumulated, which will greatly add to the importance of the annual session, and give zest to its deliberations.

Without enumerating the various considerations which at this particular time seem to call for zealous and energetic action, each member will doubtless see the necessity of being at his post.

Several distinguished members of the profession, outside the State, have signified their intention to be present; and many more will be invited.

The well known hospitality of our brethren in the beautiful "Star City," and the unusual interest which the meeting promises, we trust will prove sufficient inducement to call out every member of the Association.

To those of the profession in the State who have not yet joined our Association, as well as all physicians who feel a friendly interest in our specialty, we extend a most cordial invitation to be present and join in our deliberations.

Our object being to elevate the standard of professional excellence, and to harmonize and consolidate the interests of the Craft, with the ultimate view of benefiting alike the practitioner and the patient, we can but believe that it will prove greatly to the advantage of all interested to come promptly forward and aid in the good cause.

All those who receive this Circular are requested to extend the above invitation to all respectable Physicians and Dentists in their vicinity, or furnish a list of them with name, title and address, to the undersigned, Box No. 327, Terre Haute, Ind.

S. B. Smith,
Rec. and Cor. Sec. Ind. State Dental Association.

### CONTENTS.

### DENTAL REGISTER,

### OF THE WEST.

### Issued Monthly, at $3 a year in advance.

DEVOTED TO THE INTERESTS OF THE DENTAL PROFESSION.

**Edited by J. TAFT and GEO. WATT.**

The Volume commences in January and closes in December.

The Register being issued monthly is always fresh, therefore indispensable to the practitioner who desires to keep posted up with the new inventions and improvements which are daily developed. Neither expense nor effort will be spared to give value and variety to its contents. Articles will be illustrated with well executed engravings, whenever they will add to the interest or assist in explanation.

Its extensive circulation and frequency of issue makes it the BEST DENTAL ADVERTISING MEDIUM in the United States.

☞ CONTRIBUTIONS SOLICITED.

Address Original Communications to **Geo. Watt, Xenia, O.**

Exchanges to **J. Taft**, or **Dental Register, Cincinnati, O.**

Business Communications, to

**JNO. T. TOLAND, Publisher and Proprietor,**
38 West Fourth Street, Cincinnati, O.

Communications must reach the Editor as early as the 15th, to insure insertion in the next issue.

